# The use of contact lenses in patients with prior bleb-forming glaucoma surgery

**DOI:** 10.1186/s12886-025-04103-x

**Published:** 2025-05-06

**Authors:** Si Jie Tang, Timothy Do, Melissa Barnett, Kaaryn Pederson, Michele C. Lim

**Affiliations:** 1 School of Medicine, University of California, Davis, CA United States; 2 Department of Ophthalmology & Vision Sciences, University of California, Davis, CA United States; 3 Ernest E. Tschannen Eye Institute, 4860 Y Street, Suite 1E, Sacramento, CA 95817 USA

**Keywords:** Contact lens, Glaucoma, Glaucoma surgery, Trabeculectomy, Glaucoma drainage device

## Abstract

**Background:**

This study aims to investigate the success of contact lens (CL) wear in patients who have had bleb-forming glaucoma surgeries and to assess the rate of CL related complications.

**Methods:**

Patients who received any type of CL services at an academic center and who had a bleb-forming glaucoma surgery were identified by billing records over a 15-year period. Patients were included if they had CL fitting after bleb-forming surgery with follow-up ≥ 1 year. Information regarding patient demographics, type of bleb-forming surgery, type of CL, best corrected visual acuity (BCVA), length of follow-up, reasons for failure, and complications related to CL wear was collected. The primary outcome measure was successful CL wear for ≥ 1 year. Secondary outcome measures included complications, type of CL in success groups, reasons for CL failure, and visual acuity (VA).

**Results:**

39 eyes of 32 patients met the inclusion criteria (age, 6 months to 81 years). 20/39 eyes (51%) had successful CL wear for ≥ 1 year. No difference existed between the proportion of trabeculectomy or glaucoma drainage devices (GDD) in the CL success versus failure groups. Among the eyes that successfully wore CL, 5/20 (20%) had complications which included failed bleb, corneal edema, keratoconjunctivitis sicca, filamentary keratitis, corneal irritation, punctate epithelial keratitis, and epithelial abrasion. Within the CL failure group, one eye (1/19) developed an acute iritis directly related to CL wear. Rigid gas permeable lenses were more prevalent in the CL success group, whereas non-impression fitted scleral lenses were more prevalent in the CL failure group. At 1 year, no difference existed in BCVA for eyes that succeeded in wearing CLs and for those who did not.

**Conclusion:**

More than half of individuals with bleb-forming glaucoma surgeries were able to continue CL wear 1 year after fitting, and rigid gas permeable lenses were the most common type of lens in the CL success group. Keratopathies were the most common type of complication recorded.

## Introduction

Despite the emergence of minimally invasive glaucoma surgeries, bleb-forming surgeries such as trabeculectomy and glaucoma drainage devices (GDD) are still important for the treatment of moderate to advanced severity of glaucoma [1]. In addition, newer, bleb-forming, less invasive glaucoma surgeries such as the XEN^®^ Gel Stent and Preserflo™ MicroShunt are emerging [2]. The disadvantages of bleb-forming surgeries are postoperative infection both in the short and the long-term [3]. Bleb-related infections can be a visually threatening complication that can lead to endophthalmitis, with a high risk of vision loss even with early detection and management [3]. The use of contact lenses (CLs) can increase the risk of ocular infection, especially in patients who have poor CL hygiene [4]. Other presumed risks of contact lens wear in these patients are corneal ulcer, damage to the bleb tissue, and bleb leaks [5]. Based on these potential problems, CLs are traditionally discouraged in patients who have had bleb-forming surgeries. However, many instances exist in which patients are CL dependent, and such examples include the presence of high or irregular corneal astigmatism, high refractive error, keratoconus, and eyes that are post-corneal transplantation [6].

Various types of CLs include soft, rigid gas-permeable, hybrid, and scleral lenses. CL complications can range from keratoconjunctivitis, corneal abrasion, keratitis, corneal neovascularization, and endothelial changes [7]. Complications related to contact lenses are generally common yet mild and can be easily managed with only rare occurrences of major complications [8, 9].

In cases where the benefits of CL wear in an eye with a bleb-forming surgery outweighs the risks, it is typically recommended that patients begin CL wear 3–12 months after surgery [10]. To avoid interference with the normal functioning of trabeculectomy blebs and GDDs, rigid CL can be customized with a lens notch or impression-based device to limit the interaction of the lens with the bleb or device [11]. Nevertheless, CL fitting can be challenging because of the presence of the bleb, tube, or patch graft at the limbus, which could increase the risk of complications such as corneal ulcer and blebitis [12]. Our study’s aim is to determine the success rate of CL wear in patients who have had bleb-forming glaucoma surgery. The secondary aims are to report the rate of CL-related complications, to compare CL success rate between different types of lenses, and to compare visual acuity (VA) outcomes between patients who are and are not successful with CL wear.

## Methods

### Data collection

A retrospective chart analysis was conducted at a tertiary-level academic health organization and the study was determined exempt from full review by the Human Subjects Review Committee at the University of California, Davis. All research adhered to the tenets of the Declaration of Helsinki.

Patients who received CL services and who had bleb-forming glaucoma surgery were identified by billing records over a 15-year period. Patients of all ages were included in the study if they were fit with a contact lens after bleb-forming surgery and had a follow-up visit of at least 1 year at the time of data collection. The exclusion criteria were as follows: did not have bleb-forming surgery, only had CL fitting before bleb-forming surgery, chose not to have CL-fitting at the time of appointment, lost to follow-up without failing CL wear before 1 year, fitted for occlusive lenses, or had CL-fitting only after removal of bleb-forming surgery. Data collected from the medical records included demographics, type of bleb-forming surgery, type of CL, visual acuity (VA), length of follow-up, reasons for failure, and complications related to CL wear. The definition of success was the ability to wear a CL in the operated eye ≥ 1 year after fitting. Complications were not a criterion for failure unless it resulted in the cessation of CL wear.

### Visual acuity

All Snellen values obtained from each visit were converted to logMAR values. The baseline logMAR value was calculated as the average of two clinical encounters that were 4–6 weeks prior to the CL fitting visit. If there was only one encounter in this time period, only that single visit was used as the baseline. Baseline VA was selected based on the lowest logMAR value from either the patient’s manifest refraction, spectacle, CL (if they were prior CL wearers), or uncorrected vision. Pinhole VA, if available, was used for the lowest logMAR if only uncorrected vision was obtained during that visit. VA data was collected on the day of CL fitting, at the 1-month (20–40 days window), 6-months (5–7 month-window), 1-year (8–14 month-window), and 2-year (20–28 month-window) follow-up visits. VA data collected at fitting and follow-up visits included logMAR value from spectacles, CLs, CL spherical over-refraction (SOR), or manifest refraction.

Low VA such as those with “Hand Motion” and “Count Fingers”, were converted to Snellen ratios of 20/4000 and 20/1500 respectively [13, 14]. Snellen VA for 3 patients were unable to be obtained due to their young age.

### Statistical analysis

Fischer’s exact tests for type of CL, type of surgery, and complications were conducted in R (R Foundation for Statistical Computing, Vienna, Austria). All other analyses were performed on Prism GraphPad Version 9. Chi-squared tests were used to assess categorical demographic data. Unpaired t-tests were used for the VA analyses between CL success and failure groups based on the assumption that logMAR values follow a Gaussian distribution. Wilcoxon tests were used for the CL versus spectacle analyses on the day of the CL fitting. Kaplan Meier analysis was performed for the length of time of CL wear.

## Results

### Patient demographics

53 patients who had bleb-forming glaucoma surgery and CL services were identified through billing records. 21 patients were excluded from analysis as described in the methods of the study. 39 eyes of 32 patients met pre-defined inclusion criteria and were included in the study. Patient demographics are shown in the Table 1 and no significant differences in demographic information existed between CL success and failure groups. Patients in the CL success group had a mean and SD follow-up time of 886 ± 285 days. Those in the CL failure group had a mean and SD follow-up time of 134 ± 114 days before failing contact lens use.

### Contact lens wear success rate

20/39 (51%) eyes and 17/32 (53%) patients had successful CL wear for at least 1 year.

The average CL wear-time in the success group was 9.65 ± 3.20 h per day.

### Type of bleb-forming surgery

**Fig. 1:**
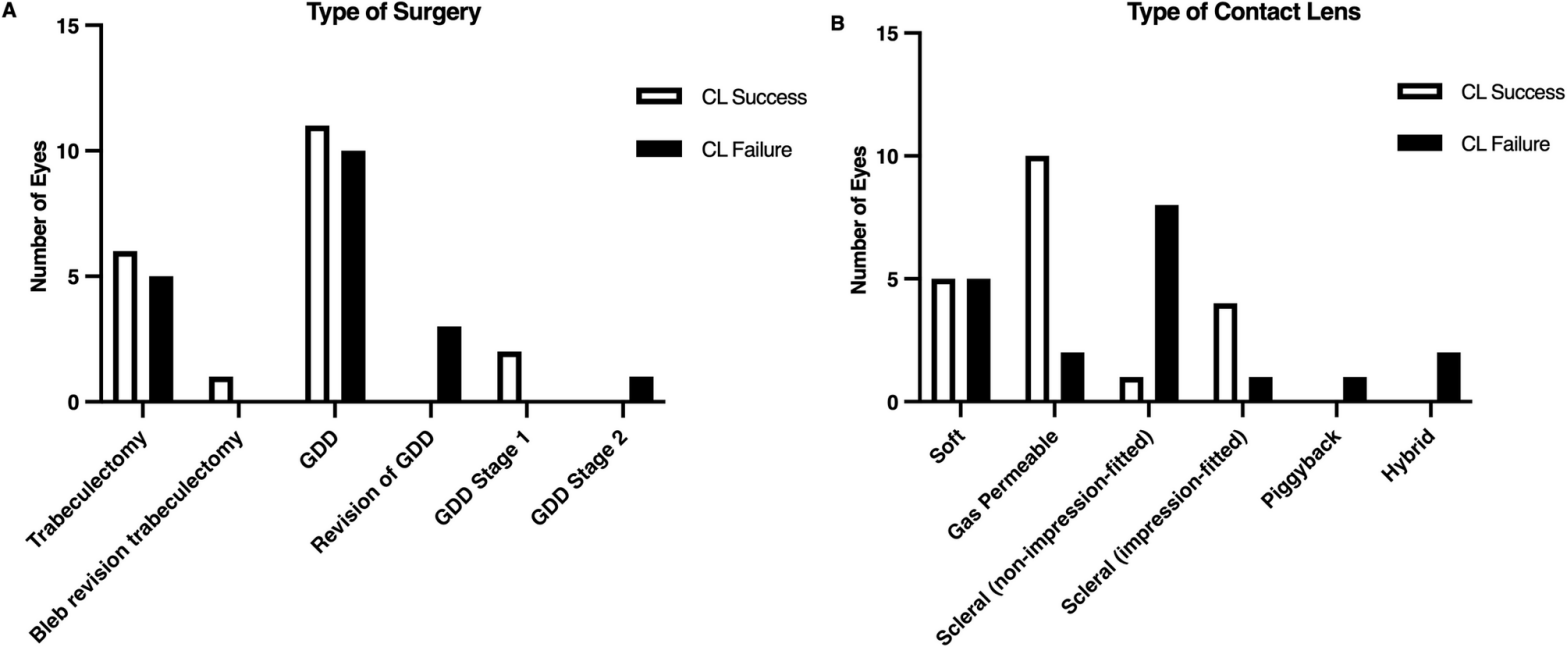
**A** and **B**. Type of glaucoma surgery and CL prescribed in the CL success and failure groups. **A**) Type of glaucoma surgery in the success versus failure groups (*p* = 0.244, *N* = 20 and 19). Some patients had GDD surgery performed in two stages. For those patients, CL-fitting that was performed after stage 1 (plate implanted but tube not placed in anterior chamber (AC)) or after stage 2 (tube subsequently placed in AC) were categorized separately. **B**) Type of CL worn in the success versus failure groups (*p* = 0.00313, *N* = 20 and 19). CL– Contact lens

Figure 1A presents the types of bleb-forming glaucoma surgeries performed for each eye. The number of each type of bleb-forming surgery in the CL success and failure groups was not significantly different (*p* = 0.244; *N* = 20 eyes in the success group and 19 eyes in the failure group). Some patients had GDD surgery performed in two stages. In stage 1 surgery, the GDD plate is sewn onto the eye, but the tube is not placed in the anterior chamber. This is usually followed by a stage 2 surgery in which the tube is then placed in the anterior chamber. For those patients, CL-fitting that was performed after stage 1 or after stage 2 were categorized separately.

### Types of lenses

Figure 1B shows CLs prescribed to eyes in the CL success and failure groups. A significant difference existed in the types of CLs used between the CL success and failure groups (*p* = 0.00313; *N* = 20 and 19 eyes). Of note, scleral lenses were further divided into non-impression-fitted versus impression-fitted. Within the non-impression-fitted scleral lens group, several were customized (the addition of a MicroVault™ to avoid the tube, a notch, or the use of a small scleral diameter).

### Gas permeable lenses versus non-impression-fitted scleral lenses

Eyes with gas permeable CL were more likely to succeed than to fail (10/12 (82%)), whereas eyes that were prescribed non-impression-fitted scleral lenses were more likely to fail than to succeed (1/9 (11%)) (Fig. 1B). Of those with non-impression-fitted scleral lenses, 5/9 (56%) eyes were fitted with customized scleral CLs and 4/9 (44%) eyes were fitted with non-customized scleral lenses. The only eye that successfully wore the non-impression-fitted scleral lenses for at least a year was fitted with a non-customized lens.

### Impression-fitted scleral lenses

5/39 (13%) eyes were prescribed impression-fitted scleral lenses (EyePrintPro, Advanced Vision Technologies, Lakewood, CO). Within this subgroup, 4/5 (80%) eyes met the CL success criteria. The one individual who did not tolerate the impression-fitted scleral lens discontinued their use of scleral lens wear due to anxiety with lens insertion.

### Reasons for failure

**Fig. 2:**
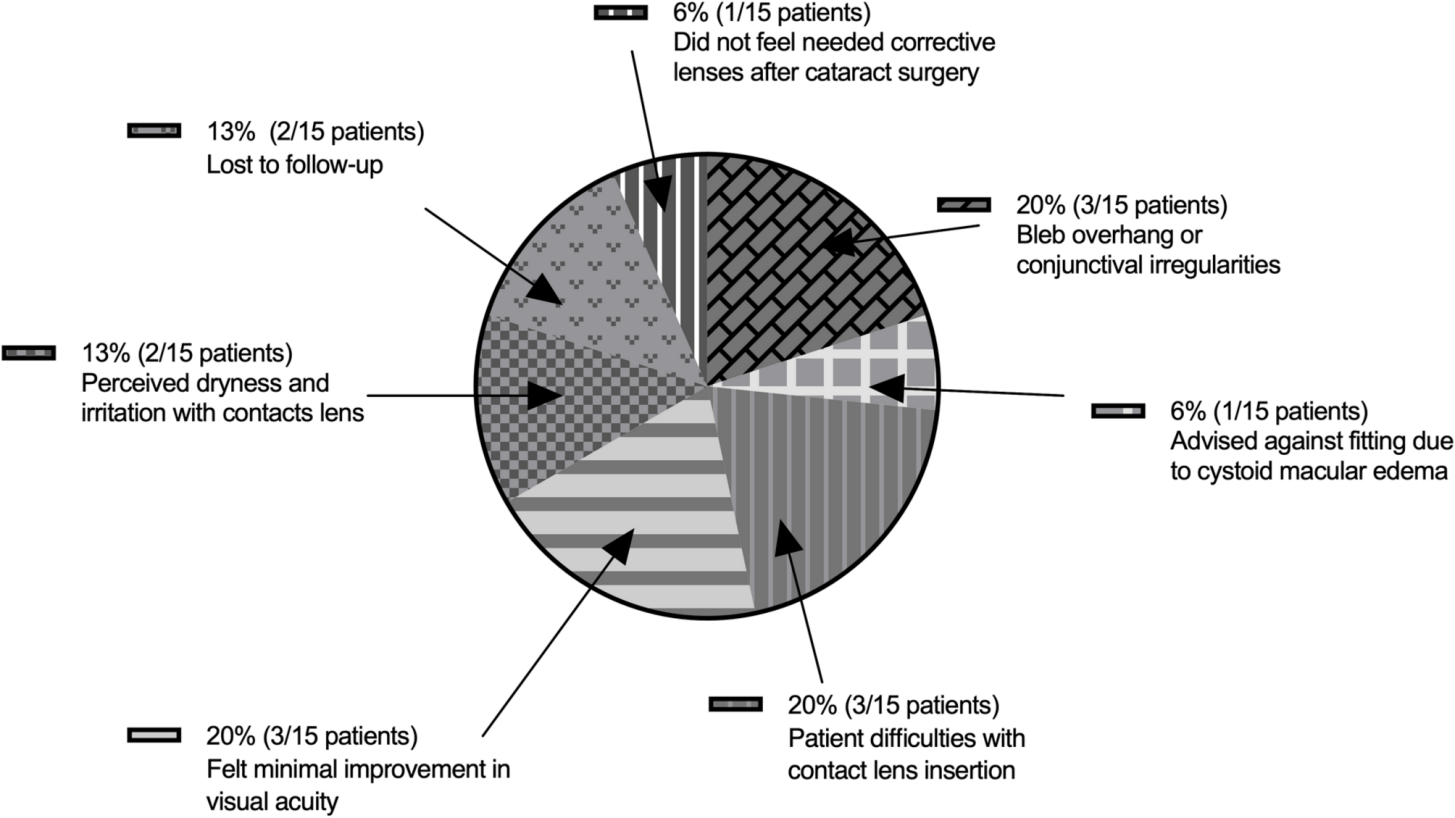
Reasons for CL wear failure. Reasons for CL failure by proportion (*N* = 15 patients)

**Fig. 3:**
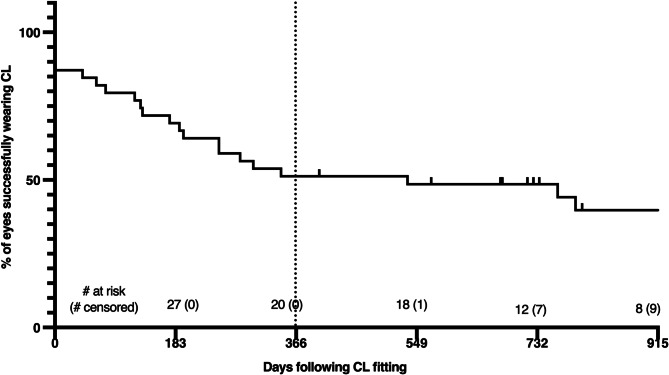
Kaplan-Meier survival analysis of CL wear following CL fitting. CL– Contact lens

Reasons for failure within one year after CL fitting are grouped in Fig. 2A and are reported per patient rather than per eye because some of the reasons were based on patient capability or were psychosocial. 15/32 (47%) patients failed CL wear within a year. Within our sample, the three most common reasons for discontinuation were due to difficulties with inserting CL (3/15 (20%)), patients feeling that there was minimal improvement in VA with CL (3/15 (20%)), and the presence of a bleb overhang or conjunctival irregularity (3/15 (20%)). The Kaplan-Meier survival analysis in Fig. 3 shows the length of time from CL fitting to discontinuation for all patients. Most discontinuation of CL wear occurred within one year of CL fitting, and only 3 patients discontinued CL after 1 year: 2 patients stated a low motivation to continue using CL and 1 pediatric patient who was uncooperative with the insertion of lenses by the guardian.

### Complications

Complications were defined as objective findings from the ocular exam. Of the CL success group, 5/20 (20%) eyes experienced complications. Within the eyes that experienced complications, 4/5 (80%) eyes each had one complication that occurred during CL wear (failed bleb (1/20, 5%), corneal edema (1/20, 5%), keratoconjunctivitis sicca (1/20, 5%), and filamentary keratitis (1/20, 5%). The fifth eye (1/20, 5%) had multiple complications that occurred several times over 14 years of CL wear (punctate epithelial keratitis and epithelial abrasion).

Of the CL failure group, only 1/19 (5%) eye had a complication noted as an acute iritis potentially caused by CL wear; however, the ultimate reason for CL discontinuation was due to perceived dryness and irritation. No difference existed in the number of complications between the CL failure and success groups (*p* = 0.182; *N* = 20 and 19 eyes).

For both groups, none of the complications resulted in cessation of CL wear.

### Visual acuity

At baseline, no difference in VA existed between the two groups (Table 1).

**Table 1:** Patient Demographics

Category	Group	CL Success (*N* = 17 patients, 20 eyes) (%)	CL Failure (*N* = 15 patients, 19 eyes) (%)	*P* value
Age Years Mean(SD) [range]		52.40(26.81) [6 months to 81 years old]	50.32 (17.77)	0.7767
Sex N (%)	Male Female	7 (42) 10 (58)	8 (53) 7 (47)	0.7235
Race	Caucasian African American Asian Other Declined to State	10 (59) 2 (12) 3 (18) 2 (12) 0 (0)	8 (53) 1 (7) 1 (7) 3 (20) 2 (13)	0.4562
Study Eye N (%)	Right Left	6 (30) 14 (70)	10 (53) 9 (47)	0.2003
Previous CL wearers (%)		14 (82)	8 (53)	0.1284
Glaucoma Diagnosis N (%)	Primary open angle glaucoma Chronic angle closure glaucoma normal tension glaucoma juvenile open angle glaucoma traumatic glaucoma uveitic glaucoma steroid induced glaucoma aphakic glaucoma Childhood angle-closure glaucoma low tension glaucoma Aniridic glaucoma Glaucoma following Keratoprosthesis End-stage glaucoma of childhood ICE syndrome with secondary glaucoma Anterior Segment Dysgenesis	6 (30) 1 (5) 2 (10) 0 (0) 1 (5) 2 (10) 0 (0) 1 (5) 1 (5) 3 (15) 1 (5) 1 (5) 0 (0) 0 (0) 1 (5)	11 (58) 1 (5) 0 (0) 2 (11) 0 (0) 0 (0) 1 (5) 0 (0) 0 (0) 1 (5) 1 (5) 0 (0) 1 (5) 1 (5) 0 (0)	0.2863
Lens Status N (%)	Aphakic Crystalline Lens Posterior Chamber Intraocular Lens	5 (25) 6 (30) 9 (45)	2 (11) 6 (32) 11 (58)	0.4817
Number of Prior Ocular surgeries Mean(SD)		1.24	1.93	0.2426
VA Baseline (LogMAR)	*N* = 17* eyes Success vs. 19 eyes Failure	0.7617 ± 0.5985	0.5973 ± 0.6014	0.4178
Incision Type N	Fornix Based Limbus Based Not Available	6 13 1	7 9 3	0.4107

*Snellen VA was unable to be obtained for 3 of the 20 patients in the success group due to young age

### Visual acuity on day of contact lens fitting

**Fig. 4:**
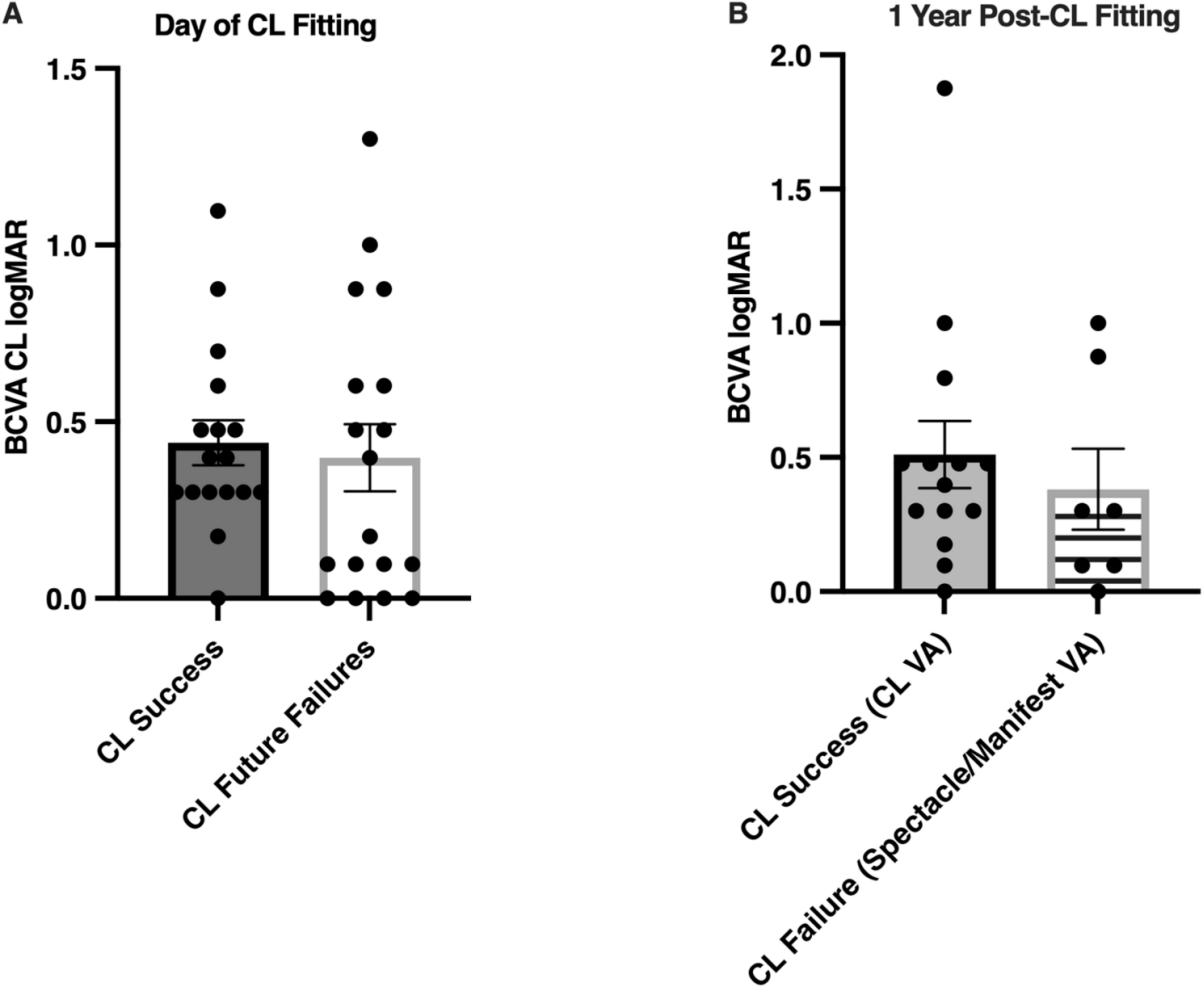
CL vision on the day of CL fitting and at 1 year follow-up visit. **A**) BCVA (logMAR) with CL did not differ between success and future failure groups on the day of CL fitting (*p* = 0.721; *N* = 17 and 18 eyes). **B**) BCVA (logMAR) 1 year after CL fitting did not differ between success and failure groups (*p* = 0.560; *N* = 14 and 7 eyes). All values are mean ± SEM. CL– contact lens, BCVA - best corrected visual acuity, SOR– spherical over refraction

We were interested in knowing whether VA with CL wear was different between patients in the CL success group on the day of contact lens fitting versus patients who eventually did not successfully wear CL for 1 year or more. Therefore, VA data while wearing CL on day of fitting were collected for both cohorts. For patients with available VA data, no significant difference existed between these two groups (Fig. 4A) (*p* = 0.721; *N* = 17 and 18 eyes). A sub-population of patients at this time point had both CL and spectacle VA data available (7/17 (41.2%) of eyes in the CL success group and 8/18 (44.4%) of eyes in the CL failure group). Both success and failure groups had better VA when wearing CL than when wearing spectacles (CL Success: *p* = 0.0310; CL Failure: *p* = 0.0310).

### Visual acuity at 1 year

At one year, patients in the CL failure group were unable to wear their CL, and we were interested in comparing VA between groups at this time. VA for the success group was assessed with CL wear (CL and CL SOR), and VA for the failure group was assessed without CL (spectacle and manifest refraction), and it did not differ significantly (Fig. 4B) (*p* = 0.560; *N* = 14 and 7 eyes).

## Discussion

Patients with glaucoma may be dependent on CLs for good vision for specific reasons including high refractive error, corneal surface irregularity or severe dry eye disease. CL wear may be challenging for individuals who have had prior bleb-forming glaucoma surgery due to fitting issues, the potential for damage to the bleb, or the risk of infection. Therefore, we aimed to determine the rate of success of CL wear in patients who have had prior bleb-forming glaucoma surgeries to help us decide whether it is feasible in this patient population.

Our study found that the rate of successful CL wear, defined as having worn CL for one or more years, was 51% of eyes which was slightly lower than that found in the literature. In our cohort, eyes that were fitted with gas permeable lenses were more likely to successfully wear CL for one or more years compared to other types of lenses, including non-impression fitted scleral lenses. A previously published study demonstrated that the success rate of CL wear after trabeculectomy was 85% after a median duration of 11.5 months. However, only rigid gas permeable lenses were worn without comparison to other types of lenses [15]. Among patients without bleb forming surgeries, studies reported success rates of 74% among both neophyte CL wearers and 77% among those refitted after a lapse in CL wear [16, 17].

In our study, complications were predominantly ocular surface findings such as keratoconjunctivitis and keratitis. In a normal population, CLassociated keratitis was estimated to be 2 to 5 per 10,000, and the majority were related to bacterial infections, but dry eyes remain the highest complication associated with CL in normal CL-wearers [18, 19]. Other complications experienced by normal CL-wearers but not by those in our study were giant papillary conjunctivitis, presence of papillae, and neovascularization [8].

Interestingly, the type of glaucoma surgery did not seem to influence the success or failure of CL wear in our study. The bleb location differs between these surgeries: in trabeculectomy, the bleb forms closer to the limbus, whereas in comparison, the GDD bleb is approximately 8 to 9 mm posterior to the limbus. One might speculate that the CL fit and tolerance would be much more difficult with post-trabeculectomy eyes, but our study did not support this. Nonetheless, fitting a CL with a bleb at the limbus can be challenging and anything that can be done to mitigate its size should be considered. For example, evidence does suggest that limbus-based conjunctival incisions vs. fornix-based lead to higher, more avascular blebs [20]. Different methods of mitomycin-C application (injection vs. sponge) may also influence bleb morphology. A recent meta-analysis of these techniques suggests that injecting MMC leads to a more diffuse bleb with lower height [21]. Trabeculectomy surgeons employ nuances in technique to aim for the ideal bleb which is minimally vascularized, low profile, and diffuse. These are characteristics that also benefit CL fitting.

We did not find previous studies that compare CL success in post-trabeculectomy eyes to post-GDD eyes. However, case studies are available that illustrate modifications that can improve the success rate of CL wear in eyes with GDD. There has been a growing number of patients using PROSE (Prosthetic Replacement of the Ocular Surface Ecosystem) and other customizable scleral lenses which are difficult to fit in glaucoma patients [22, 23]. Scleral lens wear in patients with a GDD implant is carefully co-managed by a lens specialist and by an ophthalmologist due to the potential of tube compression, resistance to aqueous flow, or inflammation leading to tube erosion [23]. In some cases, landing zones of the scleral lens may require customization to prevent tissue compression or inflammation [23]. In a single center study, three of five eyes were reported to be successfully fitted with PROSE lenses, yet two of those successes were still met with difficultly due to the persistence of bubbles and impingement of the tube or bleb [24]. Tanhehco et al. [25] published a small case study of the PROSE lens in eyes with bleb-forming surgery. The authors described conjunctival erosion over a GDD tube in three out of four patients in whom a PROSE lens was utilized. After modification of the lens with a “scalloped edge”, the CL wear was successful with no further erosions [25]. Nguyen et al. [26] described a case series in which the scleral patch graft in 3 eyes with GDD prevented successful PROSE lens wear. Both studies included cases in which the GDD tube was placed through the pars plana, and this posterior tube insertion location allowed for successful fit and wear of the scleral lenses [25, 26]. Another technique that may help lens fit is a longer scleral tunnel for the GDD tube which may either preclude the need for a patch graft or allow for more posterior placement [27]. This would make CL fit much easier by allowing for tube entry farther from the limbus. Our study adds to current knowledge with a comparison of CL success and failure between eyes with trabeculectomy and GDD surgeries.

Our study offered a comparison of CL wear among patients using different types of lenses. The CL success group were more frequently prescribed rigid gas permeable CLs. We did not find prior studies comparing CL lens wear in glaucoma patients with bleb-forming surgeries, but studies assessing rigid gas permeable lens wear do exist. In a study with 15 patients, the rate of success was 85% for patients with rigid gas permeable CLs following trabeculectomy [15]. Similarly, in our study 83% were in the success group. A prior publication in a general CL-wearing population showed that fewer complications are associated with small diameter rigid-gas permeable CLs compared to soft CLs, which could be beneficial to patients with bleb-forming surgeries [8]. Rigid-gas permeable CLs are smaller in diameter than soft CLs and scleral lenses, and they may move less, therefore reducing possible interaction with the surgical bleb.

In our study, the CL failure group was more frequently prescribed non-impression fitted scleral lenses. Many patients find that scleral lenses are difficult to wear given their larger diameter and often experience challenges with insertion [28]. Long-term studies of scleral lens wear without a surgical bleb (> 2 years follow-up), demonstrate that 25% of patients eventually discontinue lens wear, typically due to a lack of motivation, discomfort, or limited improvement in visual acuity [29]. These issues were also common reasons for failure within our cohort.

Advances in CL technology have allowed for impression-fitted scleral lenses for patients with glaucoma. Impression-fitted scleral lenses utilize impression molds or digital scans of the ocular surface to allow for unique fitting that can accommodate for the glaucoma drainage device implant and for the blebs formed from glaucoma surgery. The lenses are then 3-D printed to create a completely customized lens. In our cohort, impression-fitted scleral lenses demonstrated promising success rates compared to traditional scleral lenses, though the sample size was limited. The ability to customize a scleral lens through an impression and 3-D scan may explain its increased success rates. Although the number of eyes using this type of scleral lens in this sample was low, they were well tolerated among those that used it. The only patient who discontinued impression-fitted scleral lens wear did so due to anxiety with inserting scleral lenses rather than intrinsic complications with the lens itself.

Finally, there was no difference in VA between the CL success and failure groups on the day of fitting and at 1 year follow-up. We must consider that other dimensions of improved vision exist beyond the VA metrics measured by the Snellen chart, which is used to assess clarity or sharpness of central vision [30]. CLs provide benefits over spectacles such as a wider field of view and improved optical quality [31]. Patients with irregular astigmatism or high myopia gain these visual benefits, and for the latter group, contact lenses can help maintain true image size compared to spectacles [32]. CLs have also been found to be superior to spectacles for quality of life [33].

One additional point to note is that patient selection for CL following bleb-forming surgery is especially critical for successful CL wear. Consideration for the presence of ocular surface disease preoperatively as well as during postoperative healing may help in determining whether a patient may be a candidate for CL [34]. Additional considerations for determining whether a CL can be successful in this population of patients are the maturity and location of the bleb, results of the trial CL fitting, the patient’s own lens hygiene and comfort with putting on CL, and the patient’s availability to follow-up with the patient’s ophthalmologist and lens specialist following fitting [35].

In summary, this study demonstrates that more than half of our study population was able to continue CL wear 1 year after fitting, and rigid gas permeable lenses were more common in the success group. The incidence of serious complications within one year after CL fitting was low; however, the sample size and follow-up time precluded definitive conclusions regarding long-term safety. The limitation of this study includes our small sample size and its retrospective nature. Further studies are needed to investigate other facets of vision, including contrast sensitivity and peripheral vision, to assess whether CLs can provide improved visual outcomes. These results will provide a framework for better understanding which type of CLs are best suited for patients with bleb-forming glaucoma surgeries. Insights from these studies could also contribute in helping to engineer the next generation of CLs for this specific patient population.

## Data Availability

The datasets used and/or analysed during the current study are available from the corresponding author on reasonable request.

## Abbreviations

CL: Contact Lens
VA: Visual Acuit
BCVA: Best Corrected Visual Acuity
GDD: Glaucoma Drainage Devic
SOR: Spherical Over Refraction Value

## References

[1] Brandão LM, Grieshaber MC. Update on minimally invasive Glaucoma surgery (MIGS) and new implants. J Ophthalmol. 2013;2013:p705915. 10.1155/2013/705915 PMC3863473 24369494

[2] Scheres LMJ, et al. XEN(^®^) gel stent compared to PRESERFLO™ microshunt implantation for primary open-angle glaucoma: two-year results. Acta Ophthalmol. 2021;99(3):e433–40. 32909682 10.1111/aos.14602 PMC8246811

[3] Koike KJ, Chang PT. Trabeculectomy: A brief history and review of current trends. Int Ophthalmol Clin. 2018;58(3):117–33. 29870414 10.1097/IIO.0000000000000231

[4] Nau CB, et al. Demographic characteristics and prescribing patterns of scleral Lens fitters: the SCOPE study. Eye Contact Lens. 2018;44(Suppl 1):S265–72. 28617729 10.1097/ICL.0000000000000399

[5] Bellows AR, McCulley JP. Endophthalmitis in Aphakic patients with unplanned filtering blebs wearing contact lenses. Ophthalmology. 1981;88(8):839–43. 7322502 10.1016/s0161-6420(81)34941-0

[6] Jacobs DS, et al. BCLA CLEAR– Medical use of contact lenses. Contact Lens Anterior Eye. 2021;44(2):289–329. 33775381 10.1016/j.clae.2021.02.002

[7] Gurnani B, Kaur K. *Contact Lens–Related Complications*, in *StatPearls*. 2023, StatPearls Publishing Copyright © 2023, StatPearls Publishing LLC.: Treasure Island (FL).

[8] Forister JF, et al. Prevalence of contact lens-related complications: UCLA contact lens study. Eye Contact Lens. 2009;35(4):176–80. 19474751 10.1097/ICL.0b013e3181a7bda1

[9] Stapleton F, et al. CLEAR - Contact lens complications. Cont Lens Anterior Eye. 2021;44(2):330–67. 33775382 10.1016/j.clae.2021.02.010

[10] Malet F. Practical measures. Contact lenses after glaucoma surgery. J Fr Ophtalmol. 2006;29(Spec 2):49–51. 17072223 10.1016/s0181-5512(06)73956-0

[11] Fadel D, Kramer E. Potential contraindications to scleral lens wear. Contact Lens Anterior Eye. 2019;42(1):92–103. 30392894 10.1016/j.clae.2018.10.024

[12] Nguyen MTB, Thakrar V, Chan CC. EyePrintPRO therapeutic scleral contact lens: indications and outcomes. Can J Ophthalmol. 2018;53(1):66–70. 29426444 10.1016/j.jcjo.2017.07.026

[13] Bonnan M, et al. Short delay to initiate plasma exchange is the strongest predictor of outcome in severe attacks of NMO spectrum disorders. J Neurol Neurosurg Psychiatry. 2018;89(4):346–51. 29030418 10.1136/jnnp-2017-316286

[14] Schulze-Bonsel K, et al. Visual acuities hand motion and counting fingers can be quantified with the Freiburg visual acuity test. Volume 47. Investigative Ophthalmology & Visual Science; 2006. pp. 1236–40. 3. 10.1167/iovs.05-0981 16505064

[15] Pederson K. Enhance postoperative filtering bleb-induced vision difficulties with well-fitted GP contact (oxygen-permeable) lenses. Optometry. 2005;76(2):115–22. 15732628 10.1016/s1529-1839(05)70264-5

[16] Sulley A, Young G, Hunt C. Factors in the success of new contact lens wearers. Cont Lens Anterior Eye. 2017;40(1):15–24. 27818113 10.1016/j.clae.2016.10.002

[17] Young G, et al. A multi-centre study of lapsed contact lens wearers. Ophthalmic Physiol Opt. 2002;22(6):516–27. 12477016 10.1046/j.1475-1313.2002.00066.x

[18] Maier P, Betancor PK, Reinhard T. Contact Lens-Associated Keratitis-an often underestimated risk. Dtsch Arztebl Int. 2022;119(40):669–74. 35912449 10.3238/arztebl.m2022.0281 PMC9830382

[19] Waghmare SV, Jeria S. A review of contact Lens-Related risk factors and complications. Cureus. 2022;14(10):e30118. 36381898 10.7759/cureus.30118 PMC9644230

[20] Solus JF, et al. Comparison of limbus-based and fornix-based trabeculectomy: success, bleb-related complications, and bleb morphology. Ophthalmology. 2012;119(4):703–11. 22226886 10.1016/j.ophtha.2011.09.046

[21] Zheng J, Zhang A. Efficacy and safety of intraoperative injection of mitomycin C during trabeculectomy: a systematic review and meta-analysis. Int Ophthalmol. 2024;44(1):372. 39240391 10.1007/s10792-024-03291-7

[22] Pisano S. *Tailored Scleral Lens Design for an Advanced Glaucoma Patient*, in *Contact Lens Spectrum*. 2023.

[23] Walker MK, Schornack MM, Vincent SJ. Anatomical and physiological considerations in scleral lens wear: conjunctiva and sclera. Cont Lens Anterior Eye. 2020;43(6):517–28. 32624363 10.1016/j.clae.2020.06.005

[24] Duong AT, Ertel MK, Van Tassel SH. Glaucoma prevalence and Glaucoma surgical considerations in prosthetic replacement of the ocular surface ecosystem device use. Eye Contact Lens. 2022;48(2):69–72. 34608029 10.1097/ICL.0000000000000846 PMC8792159

[25] Tanhehco T, Jacobs DS. Technological advances shaping scleral lenses: the Boston ocular surface prosthesis in patients with glaucoma tubes and trabeculectomies. Semin Ophthalmol. 2010;25(5–6):233–8. 21091005 10.3109/08820538.2010.518873

[26] Nguyen AH, et al. Glaucoma surgical considerations for PROSE lens use in patients with ocular surface disease. Cont Lens Anterior Eye. 2016;39(4):257–61. 26876498 10.1016/j.clae.2016.02.002

[27] Pakravan M, et al. Ahmed Glaucoma valve implantation: graft-Free short tunnel small flap versus scleral patch graft after 1-Year Follow-up: A randomized clinical trial. Ophthalmol Glaucoma. 2018;1(3):206–12. 32672654 10.1016/j.ogla.2018.10.008

[28] Kanakamedala A, et al. Outcomes of scleral contact Lens use in veteran population. Eye Contact Lens. 2020;46(6):348–52. 31794543 10.1097/ICL.0000000000000671

[29] Barnett M, et al. CLEAR - Scleral lenses. Cont Lens Anterior Eye. 2021;44(2):270–88. 33775380 10.1016/j.clae.2021.02.001

[30] Daiber HF, Gnugnoli DM. *Visual Acuity*, in *StatPearls*. 2023, StatPearls Publishing Copyright © 2023, StatPearls Publishing LLC.: Treasure Island (FL).

[31] Vincent SJ. The use of contact lenses in low vision rehabilitation: optical and therapeutic applications. Clin Exp Optom. 2017;100(5):513–21. 28664572 10.1111/cxo.12562

[32] Demir M, et al. Assessment of aberrations and visual quality differences between myopic and astigmatic eyes before and after contact lens application. North Clin Istanb. 2015;2(1):1–6. 28058332 10.14744/nci.2015.87487 PMC5175044

[33] Pomeda AR, et al. MiSight assessment study Spain: A comparison of Vision-Related Quality-of-Life measures between misight contact lenses and Single-Vision spectacles. Eye Contact Lens. 2018;44(Suppl 2):S99–104. 28719538 10.1097/ICL.0000000000000413

[34] Mukamal R. *Navigating Surgical Blebs and Contact Lens Wear in Glaucoma Patients*, in *American Academy of Ophthalmology: EyeNet Magazine*. 2022.

[35] Samples JR, Andre M, MacRae SM. Use of gas permeable contact lenses following trabeculectomy. CLAO J. 1990;16(4):282–4. 2249347

